# Bioactive Excreted/Secreted Products of Entomopathogenic Nematode *Heterorhabditis bacteriophora* Inhibit the Phenoloxidase Activity during the Infection

**DOI:** 10.3390/insects11060353

**Published:** 2020-06-05

**Authors:** Sara Eliáš, Jana Hurychová, Duarte Toubarro, Jorge Frias, Martin Kunc, Pavel Dobeš, Nelson Simões, Pavel Hyršl

**Affiliations:** 1 Department of Experimental Biology, Faculty of Science, Masaryk University, Kamenice 753/5, 625 00 Brno, Czech Republic; saraelias@mail.muni.cz (S.E.); jana.hurychova@mail.muni.cz (J.H.); 376041@mail.muni.cz (M.K.); 2 CBA and Faculty of Sciences and Technology, University of Azores, Rua Mãe de Deus n° 13, 9500-321 Ponta Delgada, Portugal; duartetoubarro@uac.pt (D.T.); jorge.mv.frias@uac.pt (J.F.); nelson.jo.simoes@uac.pt (N.S.)

**Keywords:** *Heterorhabditis bacteriophora*, excreted/secreted products, phenoloxidase, immunity, melanization, *Galleria mellonella*, virulence

## Abstract

Entomopathogenic nematodes (EPNs) are efficient insect parasites, that are known for their mutualistic relationship with entomopathogenic bacteria and their use in biocontrol. EPNs produce bioactive molecules referred to as excreted/secreted products (ESPs), which have come to the forefront in recent years because of their role in the process of host invasion and the modulation of its immune response. In the present study, we confirmed the production of ESPs in the EPN *Heterorhabditis bacteriophora*, and investigated their role in the modulation of the phenoloxidase cascade, one of the key components of the insect immune system. ESPs were isolated from 14- and 21-day-old infective juveniles of *H. bacteriophora*, which were found to be more virulent than newly emerged nematodes, as was confirmed by mortality assays using *Galleria mellonella* larvae. The isolated ESPs were further purified and screened for the phenoloxidase-inhibiting activity. In these products, a 38 kDa fraction of peptides was identified as the main candidate source of phenoloxidase-inhibiting compounds. This fraction was further analyzed by mass spectrometry and the de novo sequencing approach. Six peptide sequences were identified in this active ESP fraction, including proteins involved in ubiquitination and the regulation of a Toll pathway, for which a role in the regulation of insect immune response has been proposed in previous studies.

## 1. Introduction

The genera *Steinernema* and *Heterorhabditis* are comprised of parasitic nematodes which live in mutualistic relationships with the bacteria *Xenorhabdus* and *Photorhabdus*, respectively. There is increasing interest in entomopathogenic nematodes (EPNs) as effective agents for the control of a variety of insect pests [1,2,3], as well as with regards to understanding the host–parasite interactions [4,5,6,7].

EPNs have four larval stages, most of which are present only inside an infected insect cadaver. The only stage that lives freely in the soil is the developmentally arrested larval stage, called the infective juvenile (IJ). EPNs use various strategies to locate a host [8]. The cruiser strategy is characterized by active searching in response to host cues [9], whereas the ambusher strategy is described as remaining in one spot while waiting for an insect. Many EPN species combine these two behaviors to increase the odds of finding a host, and therefore are intermediate in their foraging strategy [10,11]. Heterorhabditid species often use the cruiser approach, whereas in steinernematid species, higher variability of these tactics is usually described [10,12].

When the EPNs find an appropriate insect host, they invade its body through natural openings or by penetrating the cuticle [13,14]. Once they reach the insect hemocoel, IJs release mutualistic bacteria from their intestine and begin to develop into the reproductive adult stage [13].

The original assumption was that EPNs are primarily used as a vector for their mutualistic bacteria. The bacteria produce a mixture of molecules, which are responsible for the modulation of the host immune system [15,16], resulting in the death of the insect host. They can inhibit the growth of other bacteria by the production of antibiotics [17], and aid in the successful development of EPNs by serving as their food source. However, previous research confirmed that EPNs can survive [18], and in the case of the steinernematid species, even kill their host, without mutualistic bacteria [18,19,20].

The virulence of EPNs results from their ability to find a host, invade it and, in cooperation with mutualistic bacteria, overcome its immune response and kill it [21]. An important defense reaction of insects is melanization. This is a common reaction to foreign structures and pathogens; it is responsible for the formation of melanin and accompanies other immune reactions, e.g., nodulation or encapsulation [22,23]. This immune reaction is mediated by an enzyme called phenoloxidase (PO), which is commonly stored in the form of its inactive precursor, prophenoloxidase, which is converted into its active form by a cascade of serine proteases. The cascade is triggered after recognition of a non-self structure, through pathogen-associated molecular patterns which are also present on the surfaces of EPNs and their mutualistic bacteria. 

EPNs are able to penetrate into a host hemocoel and overcome its immune system. For this purpose, they produce excreted/secreted products (ESPs) with various functions. Several ESPs that are produced by *S. carpocapsae* have been identified and their function characterized, including their interaction with the host immune response [24,25,26,27,28,29,30,31,32,33]. The suppression of the host immune system is essential for successful infection and the death of the host. In *S. carpocapsae*, inhibitors of both humoral and cellular immune responses have been described. Penetration into the insect host is the first step of the EPN infection process. Genes *sc-asp113* (GenBank Accession ID. HM189211.1) and *sc-asp155* (GenBank Accession ID. HQ412766), encoding aspartic proteases, are upregulated at the beginning of the parasitic phase, and are probably involved in the disruption of the host tissue [28,29]. Additionally, the astacin metalloprotease Sc-AST (GenBank Accession ID. GU199041.1), identified by Jing et al. [34], could participate in the parasitic process. Its specific function has not been described, but this metalloprotease is upregulated during the parasitic stage of *S. carpocapsae*, which suggests it has an important role in nematode infection. Another detected protein, Sc-SP-3 (GenBank Accession ID. FJ152416.1), belonging to the chymotrypsin-like serine protease family, causes apoptosis of insect cells [26]. Chymotrypsin serine protease, identified in the ESPs of *S. carpocapsae*, can inhibit prophenoloxidase and the subsequent encapsulation of *Galleria mellonella* [24]. Similarly, the BPTI–Kunitz family of inhibitors (Sc-KU-4), produced by the same nematode species, not only causes inhibition of encapsulation, but also impairs the aggregation of hemocytes [30].

Still, little is known of the ESPs from entomopathogenic nematodes in general, and *H. bacteriophora* in particular. It was recently confirmed by Kenney et al. [35] that *H. bacteriophora* is capable of producing ESPs, and can use them to interfere with the immune system of its insect host. It is supposed that the ESP spectrum of *H. bacteriophora* could be similar to that of the known factors produced by *S. carpocapsae*. The characterization of specific molecules produced by nematodes could offer new possibilities for EPNs in field applications, as well as in improved efficacy of the previously used nematode-based pesticides. Currently, entomopathogenic nematodes are widely used in biological control; therefore, the specific identification and description of ESPs, and the virulence of EPNs, could be relevant. Alternatively, ESPs can be utilized in human medicine, because products of some entomopathogens contain compounds that can also affect human immunity [36,37,38,39] or act as antibiotics [40,41], which makes the ESPs of nematodes an interesting source of bioactive molecules with potential use in pharmacology.

In the present study, we present evidence indicating that the virulence of *H. bacteriophora* is affected by the age of the infective juveniles, and that ESPs produced by *H. bacteriophora* can inhibit PO-catalyzed melanization in *G. mellonella* larvae.

## 2. Materials and Methods

### 2.1. Insect and Nematodes

Larvae of the greater wax moth *Galleria mellonella* (Lepidoptera: Pyralidae) were reared on the artificial diet prepared according to Haydak [42], at 29 °C and in constant darkness. For experiments, larvae of VIIth instar were used.

The nematodes *Heterorhabditis bacteriophora*, strain Az148, originally isolated in Azores [43], were cultured under laboratory conditions with *G. mellonella* larvae as the optimal host. The infection of *G. mellonella* larvae was performed at 25 °C in Petri dishes containing one layer of filter paper soaked with nematode suspension. IJs emerged from the infected cadavers showing typical red coloration, were collected in a White trap [32], and stored at 11 °C in the dark and in a concentration of 25,000 IJs/mL of tap water until further use. Each batch of nematodes was used as one biological replicate, except for experiments where we followed the changes of IJs virulence in time. In the second case, the collected nematodes were stored at 25 °C in the dark and their virulence was tested in selected time points. 

### 2.2. Nematode Virulence Assay

The nematodes were used for the virulence assays at 0, 7, 14, 21, 28 and 35 days of storage. Storage of the IJs began immediately after their emergence from insect cadavers. Virulence of IJs at different time points of storage was determined by mortality assay using *G. mellonella* larvae. The larvae were put individually into the wells of a 24-well cell culture plate with a flat bottom, together with a piece of tissue paper soaked with 50 µL of tap water containing five IJs, and incubated in the dark at 29 °C. After 48 h the mortality of insect larvae was scored. The larvae were considered infected when they did not respond to prodding by blunt tweezer and their body showed typical signs of EPNs infection, i.e., loss of turgor and red coloration. For each test, 72 larvae of *G. mellonella* separated into three groups were used. The virulence assay was repeated in three independent experiments and each of them was performed with one batch of IJs stored from its emergence up to 35 days. 

### 2.3. Isolation of ESPs 

**Figure A1:**
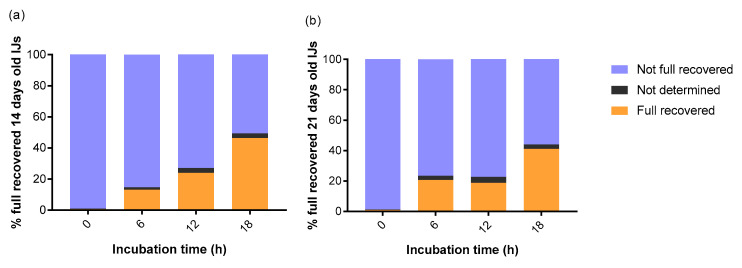

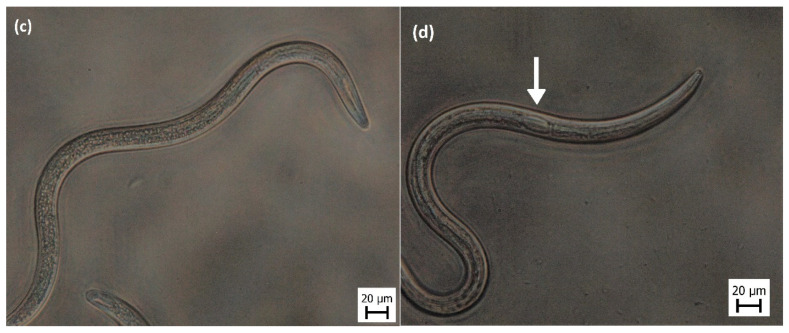
The quantification of IJs recovery during incubation with *G. mellonella* homogenate. (**a**) Percentage of fully recovered 14-day-old IJs and (**b**) percentage of fully recovered 21-day-old IJs. For each time point at least 300 IJs were counted. The full recovery was scored based on two parameters, i.e., open mouth and visible pharyngeal bulb. IJs that we could not certainly characterize as fully or not recovered were evaluated as not determined. (**c**) The representative image of the not recovered IJ can be compared to (**d**), which shows the fully recovered IJ with enlarged pharyngeal bulb (indicated by white arrow). Both (**c**) and (**d**) were observed under light microscope with 400× magnification.

ESPs were isolated from the culture of IJs stored for 7, 14, 21 and 28 days at 11 °C. The IJs were induced in vitro by adding *G. mellonella* tissue homogenate, and incubated for 18 h according to previously published protocol [26]. During the incubation of nematodes with homogenate, the activation of IJs was evaluated regularly to determine the percentage of fully recovered individuals (Figure A1). The full recovery was determined by enlarged pharyngeal bulb and open mouth [31,44], which were observed under light microscope (400× magnification). After 18 h incubation, IJs were carefully washed from insect tissues with 0.8% NaCl and transferred into Tyrode solution (0.8% NaCl, 0.02% KCl, 0.02% CaCl_2_, 0.02% MgCl_2_, 0.005% NaH_2_PO_4_, 0.1% NaH_2_CO_4_, 0.1% C_6_H_12_O_6_). After 4 h, the nematodes were separated in a 0.22-μm cellulose acetate membrane (Merck Millipore, Carrigtwohill, Ireland) to obtain ESPs solution. Subsequently, ESPs were concentrated in Amicon™ centrifugal filter units with a regenerated cellulose membrane of 5 kDa cut-off (Merck, Carrigtwohill, Ireland). The final concentration of proteins was measured by NanoDrop Protein A280 (Thermo Fisher Scientific, Wilmington, NC, USA).

### 2.4. Proteolytic Activity of ESPs

The proteolytic activity of isolated ESPs was measured using azoalbumin as substrate (Sigma, St. Louis, MO, USA). The total volume of 200 µL of the sample, containing 100 µL of ESPs at the concentration 0.1 mg/mL and 100 µL of 2% azoalbumin dissolved in distilled water, was incubated for 3 and 18 h at 37 °C, in the screening of proteolytic activity during ESPs purification and tests of age-dependent changes of ESPs activity, respectively. The reaction was stopped by adding 40 µL trichloroacetic acid (Sigma-Aldrich, St. Louis, MO, USA). The samples were kept on ice for 10 min and then centrifuged (5 min, 10,000× *g*) to remove the rest of the substrate. The absorbance of supernatant was measured at 450 nm using spectrophotometer Sunrise (Tecan, Männedorf, Switzerland). Tyrode solution was used as negative control without proteolytic activity. The activity measured in negative control ranged from around 5.47 to 0.32 U and was always subtracted from the measured values of isolated ESPs to obtain proteolytic activity of nematode products. The proteolytic activity of ESPs is represented in units (U), where one unit of proteolytic activity was defined as the amount of enzyme required to produce the change in absorbance 0.01 under the conditions of the assay.

### 2.5. Purification of ESPs

For ESPs fractionation, one milliliter of concentrated ESPs was applied in an anionic exchange column Hitrap-HQ (GE Healthcare, Chicago, IL, USA) coupled to a ÄKTA FPLC system (Amersham Biosciences, Little Chalfont, UK), equilibrated with 50 mM Tris-HCl, pH 8.8, and the protein eluted in four steps of NaCl (0.15, 0.30, 0.6 and 1 M). Fractions with PO-inhibiting activity were pooled, concentrated and applied in a size exclusion Superdex 75 HR 10/300 GL prepacked column (GE Healthcare, Chicago, IL, USA), previously equilibrated with phosphate buffer 50 mM, pH 7 and 150 mM NaCl; the proteins were eluted by the same buffer. The Gel Filtration Calibration Kit of Low molecular weight (GE Healthcare, Chicago, IL, USA) was used at the same conditions. The molecular weight of the eluted ESPs’ proteins was determined by comparison of its elution volume with those of several known calibration standards using a linear regression curve.

### 2.6. Phenoloxidase Assay

The PO activity was measured in the hemolymph of *G. mellonella* larvae. The hemolymph was collected from amputated proleg and pooled into tubes containing cold anticoagulant buffer (0.75% NaCl, 0.035% KCl, 0.021% 20 mM EDTA) in dilution ratio 1:5. The solution was centrifuged for three minutes at 1500× *g* to remove hemocytes, and the supernatant was used to assay PO activity. A volume of 40 µL of the cell-free hemolymph plasma was mixed with 20 µL of ESPs solution (50 µg/mL, final concentration) and incubated for 15 min at 25 °C. As a negative control, Tyrode solution (0.8% NaCl, 0.02% KCl, 0.02% CaCl_2_, 0.02% MgCl_2_, 0.005% NaH_2_PO_4_, 0.1% NaH_2_CO_4_, 0.1% C_6_H_12_O_6_) was used instead of ESP. After treatment with ESPs/Tyrode solution, 20 µL of 15 mM 3,4-dihydroxyphenylalanine dissolved in 20 mM Tris-HCl (pH 7.5) was added to 40 µL of treated hemolymph plasma, and the increase of absorbance at 490 nm was measured using microplate absorbance reader spectrophotometer Sunrise (Tecan, Männedorf, Switzerland) for 30 min at five minutes intervals.

### 2.7. Mass Spectrometry

The chromatographic peaks with PO-inhibiting activity were analyzed by nanoLC-MS using SciexTripleTOF 6600 mass spectrometer (AB Sciex Pte. Ltd., Framingham, MA, USA). Carbamidomethylation of samples was performed, by addition of dithiotreitol to a final concentration of 10 mM, for 30 min at room temperature. The free thiols were then alkylated with 30 mM iodoacetamide, for 30 min at room temperature. Tryptic peptides were loaded in a solution of 5% acetonitrile (ACN) and 0.1% formic acid (FA) on a BioBasic18 reverse phase column (0.18 × 150 mm, Thermo Electron, Waltham, MA, USA), and eluted in a linear gradient of 80% ACN and 0.1% FA at a flow rate of 2 µL/min. The linear ion trap was operated in data-dependent ZoomScan and MS/MS switching mode, using the three most intense precursors detected in a survey scan from 450 to 1600 *m/z*. The ZoomScan settings were as follows: 200 ms, maximum injection time; 3000 ions, zoom target parameter; and 3 microscans. Normalized collision energy was set to 35%, and dynamic exclusion was applied during 10 s intervals to avoid fragmenting each ion more than twice. Trypsin digestion, a maximum of one missing cleavage, a cysteine carbamidomethylation and the possibility of methionine oxidation were selected as conditions in the protein identification analyses. Two approaches were used to determine the sequence of peptide/protein: (i) search in UniProt protein sequences database, filtered for Nematoda (341,555 entries) using Protein Pilot Software v. 5.0 (AB Sciex Pte. Ltd., Framingham, MA, USA); (ii) de novo peptide sequencing of MS/MS by PEAKS Studio 7.5 Software (Bioinformatics Solutions Inc., Waterloo, ON, Canada), subsequently aligned with annotated proteins in the *H. bacteriophora* genome hosted in wormbase (20,964 coding genes; https://parasite.wormbase.org/Heterorhabditis_bacteriophora_prjna13977/Info/Index/; accessed on 4 June 2020).

### 2.8. Statistical Analysis

The statistical analyses were performed in software Prism (GraphPad Software, version 7.0, San Diego, CA, USA). The data from the virulence assay and enzymatic activity were evaluated using one-way ANOVA followed by Tukey’s multiple comparison test. The results from PO assays were compared using one-way ANOVA with Dunnett’s multiple comparisons test. Differences were considered statistically significant for *p* values < 0.05. The numbers of repetitions are depicted in the figure legends.

## 3. Results

**Figure 1:**
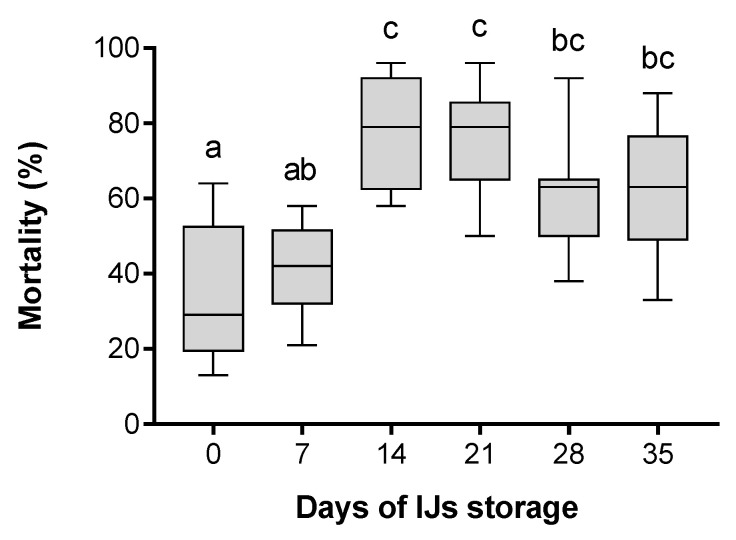
Effect of *H. bacteriophora* infective juveniles (IJs) storage time on mortality rate of *G. mellonella* larvae. Larvae were infected with nematodes stored for various numbers of days (0, 7, 14, 21, 28 and 35 days). The boxes represent the first and third quartile with the horizontal line showing the median. The whiskers depict the minimal and maximal values. Different letters above the boxes show the significant differences *p* < 0.05 (Tukey’s test), n = 9.

The mortality of *G. mellonella* larvae was tested after their infection with *H. bacteriophora* IJs stored for 0, 7, 14, 21, 28 and 35 days at 25 °C (Figure 1). The results showed that newly emerged IJs (0 days of storage after their release from the insect cadaver) were capable of invading and killing an insect host, although they caused less than 30% mortality in *G. mellonella* larvae. The mortality did not change within 1 week under storage conditions; however, with a further increase in storage time, the mortality of insect hosts significantly increased. The highest mortality of 79% ± 14.34% and 79% ± 14.26% was caused by IJs stored for 14 and 21 days, respectively. Although the mortality caused by 28- and 35-day-old nematodes was lower than the reported maximal mortality mentioned above, the difference was not significant, which shows that the tested IJs kept their virulence from 14 to 35 days after the emergence.

**Figure 2:**
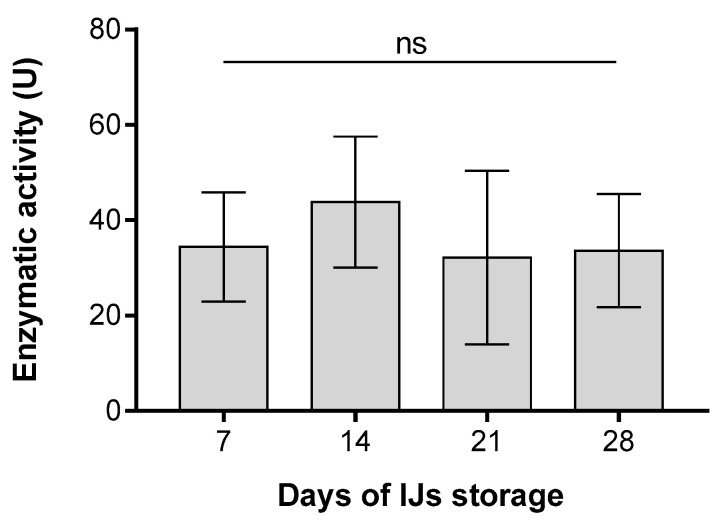
Proteolytic activity of excreted/secreted products (ESPs) produced by IJs of *H. bacteriophora* stored for various time periods before ESPs isolation (7, 14, 21 and 28 days) and measured using azoalbumin as a substrate. The y-axis represents the proteolytic activity as defined in the Material and Methods section. Columns represent mean ± SD, n = 3. No significant differences (ns) *p* > 0.05 were detected among tested groups (Tukey’s test).

Azoalbumin substrate was used to assay the proteolytic activity of the ESPs isolated from IJs of *H. bacteriophora* stored for 7, 14, 21 and 28 days (Figure 2). Tested ESPs were diluted to the same protein concentration for each assay. The proteolytic activity of ESPs was shown to be independent of the number of days of IJs storage. For all four selected time points of ESP collection, the observed mean value of proteolytic activity was approximately 36 U, without statistically significant differences among collection days. 

ESPs were collected from 14- and 21-day-old nematodes, and pooled together because of their comparable activity, to increase the volume of the sample for subsequent analyses. The 18-h long incubation of EPNs in the *G. mellonella* homogenate was selected according the percentage of fully recovered IJs, which had reached approximately 50% at this time point (Figure A1).

**Figure 3:**
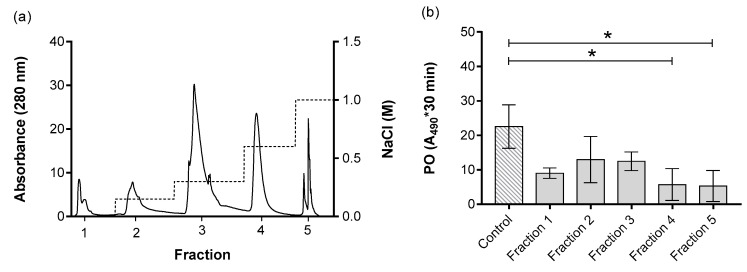
ESPs from *H. bacteriophora* separated based on their net surface charge (**a**). The left y-axis represents the absorbance at 280 nm, which correlates with the amount of proteins present in the fractions. The right y-axis represents the molar concentration of the buffer used for sample elution. The x-axis shows the fractions. (**b**) Effect of separated ESPs on phenoloxidase (PO) activity of *G. mellonella* larvae. The PO activity was measured in the absence (control) and presence of tested ESPs. The results are represented by integral of absorbance per µL of hemolymph; the enzymatic reaction was measured for 30 min. Data are presented as mean ± SD, n = 2. Note that the small number of replicates used in PO activity screen enables the retention of enough material for subsequent purifications. The asterisk * shows the significant differences *p* < 0.05 between control and specific fractions (Dunnett’s test).

The ESPs were purified based on their net surface charge, resulting in five distinct fractions, denoted as fraction 1, 2, 3, 4 and 5, which corresponded to the NaCl concentrations of 0 M, 0.15 M, 0.3 M, 0.6 M and 1 M, respectively, used for the elution of the specific fraction (Figure 3a). The effects of the purified ESPs on PO activity in the hemolymph of *G. mellonella* larvae were investigated. The assay using the PO substrate 3,4-dihydroxyphenylalanine showed the inhibition of melanization in fractions 4 and 5 (Figure 3b). Because of the observed inhibitory effect, both active fractions were subjected to a second purification, which was based on their molecular size.

**Figure 4:**
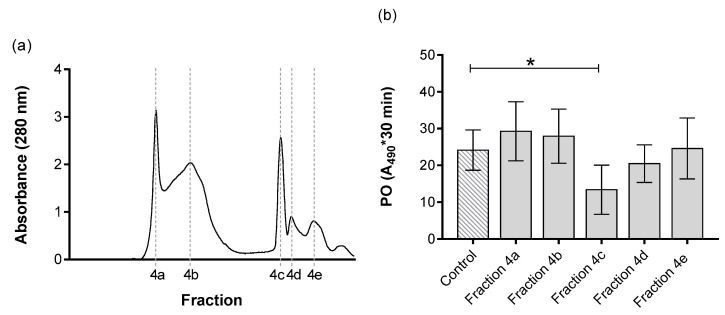
Fraction 4 from Figure 3 separated based on the molecular size (**a**). The left y-axis represents the absorbance at 280 nm, which correlates with amount of proteins present in the fractions. The x-axis shows the fractions. (**b**) Effect of separated ESPs on PO activity of *G. mellonella* larvae. The PO activity was measured in the absence (control) and presence of tested ESPs. The results are represented by integral of absorbance per µL of hemolymph; the enzymatic reaction was measured for 30 min. Data are presented as mean ± SD, n = 4. The asterisk * shows the significant differences *p* < 0.05 between control and specific fractions (Dunnett’s test).

Fraction 4 was further divided into five distinct sub-fractions, denoted as 4a, 4b, 4c, 4d and 4e (Figure 4a). The fraction 4a corresponded to molecules with the highest molecular weight of 78 kDa. In subsequent purifications, the fractions 4b, 4c, 4d and 4e exhibited a decreasing trend in their molecular weights (67 kDa, 38 kDa, 35 kDa and 27 kDa, respectively). These fractions were subjected to a PO assay to test their biological activity. The results indicated suppression of melanization in fraction 4c, with a molecular weight of 38 kDa (Figure 4b). The rest of the fractions did not cause any inhibition of melanization. 

**Figure 5:**
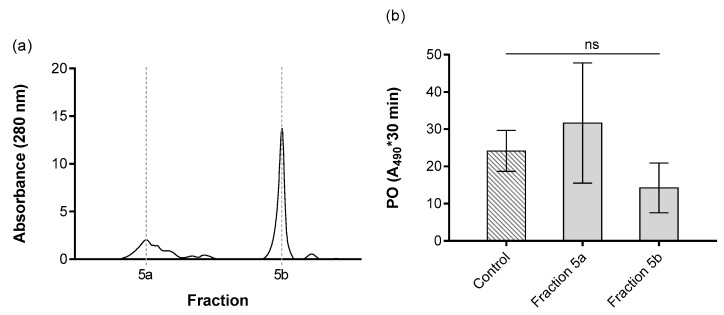
Fraction 5 from Figure 3 separated based on the molecular size (**a**). The left y-axis represents the absorbance at 280 nm, which correlates with amount of proteins present in the fractions. The x-axis shows the fractions. (**b**) Effect of separated ESPs on PO activity of *G. mellonella* larvae. The PO activity was measured in the absence (control) and presence of tested ESPs. The results are represented by integral of absorbance per µL of hemolymph; the enzymatic reaction was measured for 30 min. Data presented as mean ± SD, n = 4. Not significant differences (ns) *p* > 0.05 between control and specific fractions (Dunnett’s test).

Fraction 5 was also subjected to a second purification based on molecular size (Figure 5a). Two new sub-fractions, 5a and 5b, were obtained. The fractions 5a and 5b corresponded to the different molecular masses of 83 kDa and 37 kDa, respectively. No effect on PO activity in the *G. mellonella* hemolymph was observed for either fraction 5a or 5b (Figure 5b).

The results and details of ESP purification are summarized in Table 1. From 5 ml of total isolated ESPs, we obtained 0.71 and 0.10 mg of active proteins, during the first step of purification in fractions 4 and 5, respectively. After the second purification, we obtained 0.05 mg of active proteins in fraction 4c. From the experiments it was calculated that one nematode is able to produce approximately 0.16 ng of ESPs over 4 h.

First, we attempted to identify proteins present in chromatographic fractions that exhibited PO-inhibiting activity using the UniProt protein sequence database. Four proteins were detected in the active fraction 4c (Figure 4b) with only a single peptide match with a confidence score of 95%; thus, we assumed that the proteins were not identified. This could have been caused by insufficient representation of the annotated genome of *H. bacteriophora*, which resulted in a lack of protein sequences availability in the non-redundant protein database. Thus, we used *de novo* sequencing to identify the peptides in the active fraction. Peptide sequences obtained by *de novo* interpretation of MS/MS spectra with a quality score higher than 95% were BLAST searched with the draft genome annotation of *H. bacteriophora* [45]. The best matching *de novo* sequencing peptides are listed in Table 2 and detailed information are in Table A1a,b. In the active fraction 4c, we identified six peptides with a confidence score higher than 99%. Two of these peptides were BLAST aligned with Hba_21422 and Hba_19968, proteins implicated in ubiquitination. The transcript Hba_21422 coded for a ubiquitin protein ligase 1, homologous to the Pellino domain proteins of the parasitic nematodes *Ancylostoma ceylanicum* (acc. EPB67168), *Necator americanus* (acc. XP_013291375) and *Haemonchus contortus* (acc. CDJ86809). Hba_19968 coded for a CUE domain able to couple ubiquitin with high affinity and is homologous with proteins containing CUE domains of *H. contortus* (acc. CDJ81629) and *Dictyocaulus viviparus* (acc. KJH43919). The other four *de novo* peptide sequences identified in the active fraction 4c, BLAST aligned with different proteins: Hba_18775, homologous to ion channel proteins of *N. americanus* (acc. XP_013291760) and *Ancylostoma caninum* (acc. RCN36608); Hba_15515, homologous to the ShK toxin domain-containing proteins of *H. contortus* (acc. CDJ90282) and *A. ceylanicum* (acc.EPB74187); the Hba_18934, homologous with hypothetical proteins identified in *A. ceylanicum* (Y032_0093g2684), *N. americanus* (NECAME_00068) and *Teladorsagia circumcincta* (TELCIR_00299) and without identified Pfam domains; and Hba_20430 presented an extended winged-helix domain and showed homology with unnamed protein products of *H. placei* (acc. VDO35322), *Strongylus vulgaris* (acc. VDM68079), and *Cylicostephanus goldi* (acc. VDK71097).

First, we attempted to identify proteins present in chromatographic fractions that exhibited PO-inhibiting activity using the UniProt protein sequence database. Four proteins were detected in the active fraction 4c (Figure 4b) with only a single peptide match with a confidence score of 95%; thus, we assumed that the proteins were not identified. This could have been caused by insufficient representation of the annotated genome of *H. bacteriophora*, which resulted in a lack of protein sequences availability in the non-redundant protein database. Thus, we used *de novo* sequencing to identify the peptides in the active fraction. Peptide sequences obtained by *de novo* interpretation of MS/MS spectra with a quality score higher than 95% were BLAST searched with the draft genome annotation of *H. bacteriophora* [45]. The best matching *de novo* sequencing peptides are listed in Table 2 and detailed information are in Table A1a,b. In the active fraction 4c, we identified six peptides with a confidence score higher than 99%. Two of these peptides were BLAST aligned with Hba_21422 and Hba_19968, proteins implicated in ubiquitination. The transcript Hba_21422 coded for a ubiquitin protein ligase 1, homologous to the Pellino domain proteins of the parasitic nematodes *Ancylostoma ceylanicum* (acc. EPB67168), *Necator americanus* (acc. XP_013291375) and *Haemonchus contortus* (acc. CDJ86809). Hba_19968 coded for a CUE domain able to couple ubiquitin with high affinity and is homologous with proteins containing CUE domains of *H. contortus* (acc. CDJ81629) and *Dictyocaulus viviparus* (acc. KJH43919). The other four *de novo* peptide sequences identified in the active fraction 4c, BLAST aligned with different proteins: Hba_18775, homologous to ion channel proteins of *N. americanus* (acc. XP_013291760) and *Ancylostoma caninum* (acc. RCN36608); Hba_15515, homologous to the ShK toxin domain-containing proteins of *H. contortus* (acc. CDJ90282) and *A. ceylanicum* (acc.EPB74187); the Hba_18934, homologous with hypothetical proteins identified in *A. ceylanicum* (Y032_0093g2684), *N. americanus* (NECAME_00068) and *Teladorsagia circumcincta* (TELCIR_00299) and without identified Pfam domains; and Hba_20430 presented an extended winged-helix domain and showed homology with unnamed protein products of *H. placei* (acc. VDO35322), *Strongylus vulgaris* (acc. VDM68079), and *Cylicostephanus goldi* (acc. VDK71097).

## 4. Discussion

Although it has been known for many decades that EPNs can cause the death of an insect host [46,47,48,49], we still do not know much about the virulence factors produced by nematodes, or the mechanisms by which they interfere with the host organism. IJ is the developmentally arrested non-feeding stage of EPNs, which live free in the soil and can survive harsh environmental conditions [50]. It might take several weeks or months for IJs to locate a suitable insect host. During this time, the IJs are subjected to biological aging, and their virulence can change significantly, which subsequently affects their ability to invade and kill a host [51,52]. The negative effect of age on the movement activity of IJs was observed in *H. megidis*, in which IJs became less mobile with time [53]. The ambushing behavior of *S. carpocapsae* also decreases with the age of IJs [54]. A study focused on nematode age was performed for *Caenorhabditis elegans*, in which the older adults (>5 days old) showed lower attraction to the food odor benzaldehyde [55]. All these studies confirmed that the biological and physiological parameters of nematodes change with age; therefore, we assumed that age also positively or negatively affected their virulence.

The negative effect of age on virulence is supported by a study by Lalramliana et al. [56], who reported that the highest virulence of *S. thermophilum* and *S. glaseri* toward *G. mellonella* was observed in the first month of storage at 25 °C. After 3 and 4 months of storage, virulence began to significantly decrease, in both *S. thermophilum* and *S. glaseri*, respectively. Similar results were observed by Yoder et al. [51], who showed that *S. carpocapsae* IJs stored for less than 1 week penetrated *G. mellonella* larvae more efficiently than 4-week-old IJs. Corresponding negative effects of age on virulence have been shown in *H. megidis*, in which the virulence of IJs decreased after 2 weeks of storage [53]. Conversely, 3-month-old IJs of *S. carpocapsae* caused higher mortality in *Drosophila melanogaster* larvae than 3-week-old IJs [57]. An explanation for the differences in virulence observed in the above-mentioned studies remains unknown. However, the virulence and insect killing effectivity of EPNs can be affected by many others factors, e.g., temperature [52,56,58,59], UV radiation [60], and others. Therefore, it is necessary to always specify IJs age and storage parameters in the experimental design.

In our study, we investigated the mortality of *G. mellonella* caused by *H. bacteriophora*, correlated with the length of time the IJs were stored under laboratory conditions. Newly emerged IJs were able to kill *G. mellonella* larvae, but the highest mortality of hosts was caused by IJs stored for 14 and 21 days at 25 °C. Similar results were observed in the study of Perez et al. [61], which showed that *H. bacteriophora*’s infectivity of *G. mellonella* increased with storage time, and reached a maximum on the 14th day. However, prolonged storing time for IJs reduced their infectivity. Although the reason for the increasing trend in virulence observed in *H. bacteriophora* remains unknown, we assumed that the delay in maximal virulence may be attributed to so-called phased virulence. Hominick and Reid [62] observed that the virulence of an IJ population increased over time, and called this phenomenon phased virulence. Three phases of virulence during 4 weeks of storage were observed in *H. megidis* [63]. The early phase was characterized by low virulence, but highly active movement. The second phase was represented by the highest virulence, and IJs in the last phase exhibited decreased virulence and movement. Studies have shown that phased virulence correlates with heterorhabditid species, in which movement and virulence shift during the storage time of IJs [63,64].

EPNs can contribute to successful infection not only as a vector, which is able to invade host hemocoel and inject symbiotic bacteria, but also by the release of various products with diverse effects on the activity and survival of the host [44]. ESPs produced during the infection process are able to modulate the immune system of the host [24,26,28,30,34]; therefore, they can influence the virulence of the invading nematobacterial complex. Some of the molecules described in *S. carpocapsae* play a role in the penetration of a host (e.g., aspartic protease Sc-asp113 [29] and Sc-asp155 [28]), and some act as immune modulators (e.g., metalloprotease Sc-AST [34], chymotrypsin serine protease [24], BPTI–Kunitz family inhibitor [30] and Sc-SP-3 [26]). Furthermore, some molecules produced by nematodes can contribute to virulence through their role in the regulation of development, e.g., lamanin α [65], structurally diverse derivates of the 3,6-dideoxysugar ascarylose [66], acyl-CoA oxidases [67] and the small pheromone molecule ascaroside C11 ethanolamide [68].

According to previous studies, nematodes produce ESPs, which are essential for their virulence [24,25,26,27,28,29,30,31,32,33,35,44]. These products are vital during the invasion of IJs into the insect host [28,29], and also in the regulation of the host immune system [24,26,30,35]. However, the spectrum of ESPs contains various products that also have functions related to other biological processes, e.g., nematode development [68,69], social behavior [70,71] and nematode communication [72].

To study the ESPs, it is essential to design an assay that mimics the natural conditions of the infection, in order to obtain ESPs as similar as possible to the ones produced by free-living individuals. A key aspect is the activation of EPNs that occurs when the IJs enter an insect host. Activated IJs encompass specific characteristics, such as an open mouth [31] and expansion of the pharyngeal bulb [44]. Specifically, the activated IJs can be divided into three categories—fully, partly, and non-activated—depending on the extent to which their pharyngeal bulb is expanded. The rate of nematode activation can be affected by their attraction to a particular insect host. Alonso et al. [73] have shown that IJs activation is species-specific and host-specific; therefore, the host insect species is essential for proper activation. The intraspecific differences in infectivity have also been described in nematodes, and it was reported that various isolates of *H. bacteriophora* are differentially virulent toward *Pseudaletia unipuncta* [74].

In some studies, hemolymph has been used to activate nematodes because it can be easily obtained in desirable volumes, especially from larger insect species [35,75]. However, insect homogenate is more suitable for studies that require the same chemical composition of activation material as that of natural hosts [31]. There are some differences between in vivo and in vitro activation, because free-living IJs have to overcome the physical barriers of the hosts, such as the cuticle or gut wall, whereas during in vitro, activation these barriers are missing [73]. Chang et al. [44] suggested that in vitro activation of IJs can mimic in vivo activation. However, the specific time taken to reach the fully activated IJ form should always be experimentally verified, because IJs produce a different ESP spectrum based on the rate of their activation. These researchers observed that steinernematids were partly activated after 6 h of exposure to insect homogenate, and the number of fully activated IJs increased with time [44]. The results of Chang et al. [44] also showed similar transcriptional profiles for ESPs produced by IJs activated for 6 h in vivo and 6 h in vitro. In comparison, the 20-h long incubation of *H. bacteriophora* with insect hemolymph was used by Kenney et al. (2019) to induce the production of ESPs [35]; however, this study only focused on in vitro activation of IJs, and whether it correlates with the in vivo process remains to be clarified.

In previous studies, proteases were described as immune modulators [24,26,28,30,34], which can add to the virulence of the parasite by weakening the host immune response. Therefore, we examined the correlations between the proteolytic activity of molecules released by activated nematodes and IJs virulence. Although we observed changes in virulence with nematode aging, the enzymatic activity of ESPs remained the same regardless of nematode age. This indicated that proteases are constantly present in the spectrum of ESPs, and they do not function only as immune modulators. In this work, we showed that ESPs produced by *H. bacteriophora* have an inhibitory effect on the immune system of *G. mellonella*, similar to the effect of *S. carpocapsae* on *D. melanogaster* and *G. mellonella* [24,25,26,27,28,29,30,33,34]. *H. bacteriophora* produces a spectrum of ESPs with different functions, and some play a role in virulence [35,76]. Recently, it was described that ESPs of *H. bacteriophora* suppress the expression of the *Diptericin* gene in *D. melanogaster* [35]. This suppression could help the symbiotic bacteria *P. luminescence* to survive and overcome the insect immune defenses.

In our study, we investigated the effects of ESPs on another important insect immune reaction: the PO cascade. This is a humoral response catalyzed by the enzyme PO, which leads to the production of toxic molecules and results in the formation of melanin in reaction to non-self material [77,78,79]. Therefore, the intruding nematodes need to prevent proper activation of the host PO cascade in order to increase the success rate of the infection. Our data showed that ESPs produced by *H. bacteriophora* have the ability to inhibit the melanization of *G. mellonella*; however, we point out that the concentration of ESPs used in our experiments was higher than expected in a natural system. Melanization cooperates with other immune responses, such as the expression of antimicrobial peptides [22], which is mainly controlled by Toll and Imd pathways. In a study by Ligoxygakis et al. [80], it was shown that the melanization could be activated through the Toll pathway, but the specific link has not been described. Subsequently, it was shown that the PO cascade and the Toll pathway share a common activator: lysine-type peptidoglycan [81].

To determine the molecules involved in the process of overcoming the insect immune defense, we examined the ESPs’ PO-inhibiting activity. We identified one peptide, Hba_21422, which has a Pellino domain. Earlier, it was reported that the Pellino protein belongs to the Toll pathway regulators [82,83,84,85]. Pellino has been suggested as a positive regulator of the insect innate immune response to Gram-positive bacteria [85], but the specific mechanism of the regulation has not been described. Subsequently, in the study of Ji et al. [84], it was shown that Pellino acts as a negative regulator of the Toll signaling pathway in *D. melanogaster*. It specifically targets MyD88 and mediates its ubiquitination with subsequent degradation. By this mechanism, Pellino participates in a negative feedback loop that regulates the activity of the Toll pathway. Moreover, Pellino is a highly conserved protein and has been found in several species, including helminths, insects and humans [82,83,86]. The Pellino protein from the human parasite *Schistosoma mansoni* was shown to bind and poly-ubiquitinate human IRAK-1, a serine/threonine kinase associated with the interleukin-1 receptor in a Toll-like pathway. This regulation leads to a negative modulation of human innate immunity. Whether the Pellino produced by EPNs can positively or negatively regulate the Toll pathway would be an interesting topic for future studies.

We have also identified two other peptides associated with proteins involved in ubiquitination: an orthologue of ubiquitin protein ligase 1 (Hba_21422 transcript) and another protein with a CUE-binding domain (Hba_19968 transcript), which was shown to play a role in the interaction between ubiquitin and CUE domain-containing proteins [87]. We assume that this part of ESPs could be involved in the degradation of molecules, included those in the PO cascade. For instance, it was shown that the C-type lectin in humans, which belongs to the group of pattern recognition proteins, is negatively regulated through ubiquitination [88]. C-type lectins are also present in insects [89,90,91], and their degradation could lead to the suppression of PO cascade activity. In a study by Kud et al. [92], it was shown that the cyst nematodes of *Globodera pallida* produce an effector, which acts as a ubiquitin ligase in the interaction with the immune system of plants. The effector can suppress the effector-triggered immunity signaling, and block the recognition of pathogen-associated molecular patterns and down-stream signaling. Additionally, plant-parasitic nematodes can modulate, through ubiquitination, post-translational modifications in plants [93]. Several studies have indicated that ubiquitin probably plays an important role in the survival of nematodes during the infection of plants [93,94,95]. In the EPSs of *S. feltiae*, the ubiquitin domain was present as the third most abundant Pfam domain [44]. 

Because the EPSs contain a broad spectrum of molecules, they may be involved in a variety of immune reactions, rather than only in the regulation of melanization. The identification of other immune reactions targeted by ESPs, and a characterization of their interactions, would be a promising goal of future studies. We could thereby produce an overview regarding the interaction of nematodes with the host immune system; moreover, we could discover new possibilities for their use. 

## 5. Conclusions

The present study examined the ability of *H. bacteriophora* to regulate the PO cascade of *G. mellonella*. An important role in their immunomodulatory function is played by the ESPs produced by IJs. ESPs were isolated from 14- and 21-day-old IJs of *H. bacteriophora*, which were stored at 25 °C and caused significantly higher mortality than the newly emerged IJs, as confirmed by mortality assays using *Galleria mellonella* larvae. The ESPs were purified and subsequently tested for their PO-inhibiting activity. The fraction containing molecules with a molecular mass of 38 kDa significantly inhibited the PO cascade responsible for the melanization of the *G. mellonella* hemolymph. In this fraction, six peptide sequences were identified, including the Pellino and CUE domain proteins, which are known to participate in the regulation of Toll pathway activity and ubiquitination. We suggest that, through these mechanisms, the identified ESPs interact with the insect phenoloxidase system, and therefore modulate its immune response in favor of the nematobacterial complex. However, the exact mechanism of ESPs’ interaction with the PO system remains undescribed, and could be addressed by future studies investigating the activity of the candidate modulators identified in our study, which would be prepared in recombinant forms. 

## Figures and Tables

**Table 1:** Purification table of ESPs from *H. bacteriophora*.

	V (mL)	Protein (mg/mL)	Total Protein (mg)	Proteolytic Activity (U) ^a^	Specific Activity (U/mg) ^b^	Purifications (Folds) ^c^	% Yield^d^
Total ESP	5.00	0.67	3.37	38.01	56.48		
Fraction 1	3.96	0.02	0.10	11.91	496.25	8.79	3.57
Fraction 2	3.94	0.04	0.17	16.75	398.81	7.06	6.24
Fraction 3	3.39	0.17	0.59	14.17	81.20	1.44	25.93
**Fraction 4 ^e^**	**4.15**	**0.17**	**0.71**	**16.65**	**97.94**	**1.73**	25.26
**Fraction 5 ^e^**	**2.93**	**0.03**	**0.10**	**12.71**	**373.82**	**6.62**	5.05
Fraction 4a	1.86	0.08	0.15	29.28	374.47	6.63	11.62
Fraction 4b	3.30	0.10	0.33	27.93	281.15	4.98	14.76
**Fraction 4c ^e^**	**1.06**	**0.05**	**0.05**	**13.38**	**272.70**	**4.83**	7.29
Fraction 4d	0.42	0.01	0.00322	20.48	2667.67	47.23	1.14
Fraction 4e	1.99	0.03	0.05	24.59	952.85	16.87	3.83
Fraction 5a	2.12	0.02	0.04	34.66	2028.51	35.92	2.54
Fraction 5b	1.52	0.04	0.06	14.24	354.23	6.27	5.97

^a^ One unit (U) of proteolytic activity was defined as the amount of enzyme required to produce an absorbance change of 0.01 under the conditions of the proteolytic assay. ^b^ Specific activity was estimate as proteolytic activity of enzyme/mg protein. ^c^ Purification folds were calculated based on specific activity. ^d^ Yield is given as the percentage of proteins in the fraction. ^e^ The fractions written in bold had PO-inhibiting activity in the hemolymph of *G. mellonella*.

**Table 2:** Accurate *de novo* peptide sequencing with LC-MS/MS of active fraction 4c.

N	*de novo* Peptide Sequence ^1^	Conf Score	Hb Transcript ^2^	Identification	E Value	Homology	GO	Pfam
**1**	KPIILTNLNVPCQPK	99.0	Hba_21422	Pellino	6 × 10^−148^	*Ancylostoma ceylanicum*	Ubiquitin protein ligase	PF04710
**2**	LLAVHFTVAEGVKNKER	99.0	Hba_15515	Metridin ShK toxin	5 × 10^−148^	*Haemonchus contortus*	Toxin activity	PF01549
**3**	KQEDNEVESPPQQEVPRPR	99.0	Hba_18775	Ion channel	9 × 10^−127^	*Necator americanus*	Ion channel activity	PF07885
**4**	QKEILASTSIAQIVDRVC	99.0	Hba_18934	Hypothetical protein	6 × 10^−110^	*Ancylostoma ceylanicum*	Unnamed protein	NI
**5**	YTFKELTRQEEIVVR	99.0	Hba_19968	CUE domain protein	3 × 10^−84^	*Haemonchus contortus*	CUE domain	PF02845
**6**	MLDEVVPPAGSKLRYIC	99.0	Hba_20430	Unnamed protein	3 × 10^−16^	*Haemonchus placei*	Unnamed protein	PF02319

^1^ analysis of MS/MS by PEAKS Studio 7.5 Software ^2^ https://parasite.wormbase.org/Heterorhabditis_bacteriophora_prjna13977/Info/Index.

## Appendix A

Table A1 Peptide Summary report for fraction 4c.

**Table:** **(a)**

N	Total ^1^	%Cov (95%) ^2^	% Conf. Score ^3^	Accessions	Modifications ^4^	Cleavages ^5^	dMass ^6^	Observed MW ^7^	Observed *m/z* ^8^
**1**	2	5.703	99.00	GG5294|c2_g1_i8	Carbamidomethyl(C)@12		−0.0939	1733.8923	867.9534
**2**	2	6.227	99.00	GG7279|c2_g1_i6		missed K-N@13; missed K-E@15	−0.0881	1909.9857	956.0001
**3**	2	12.750	99.00	GG2408|c0_g1_i2		missed K-Q@1	−0.0379	2261.0654	1131.5400
**4**	2	8.531	99.00	GG16163|c66_g1_i9	Carbamidomethyl(C)@18	missed K-E@2; missed R-V@16	0.0996	2030.1835	1016.0990
**5**	2	6.522	99.00	GG15187|c0_g1_i1		missed K-E@4; missed R-Q@8	−0.0823	1909.9438	955.9792
**6**	2	5.015	99.00	GG10285|c3_g1_i4	Carbamidomethyl(C)@17	missed K-L@12; missed R-Y@14	0.0959	1947.0917	974.5531

**Table:** **(b)**

N	Theoretical MW ^9^	Theoretical *m/z* ^10^	Theoretical z ^11^	Score ^12^	Spectrum ^13^	Time ^14^	Elution Peak Width (Peptide) ^15^	Acquisition Time ^16^	Intensity (Peptide) ^17^	Precursor Intensity Acquisition ^18^	MS2Counts ^19^
**1**	1733.9862	868.0004	2	3	1.1.1.710.22	11.19	1.11	11.2966	271.28	237.27	39.1209
**2**	1910.0737	956.0442	2	3	1.1.1.715.28	11.63	0.27	11.5227	197.27	171.01	21.9854
**3**	2261.1035	1131.5590	2	3	1.1.1.762.37	13.71	0.71	13.6088	208.23	191.34	8.9114
**4**	2030.0830	1016.0490	2	3	1.1.1.724.32	11.90	0.26	11.9234	239.11	215.69	20.2243
**5**	1910.0261	956.0204	2	3	1.1.1.723.19	11.63	0.84	11.8707	253.12	182.25	20.9448
**6**	1946.9959	974.5052	2	3	1.1.1.762.25	13.62	1.63	13.5994	171.85	161.82	17.1024

^1^ Total measure of the total amount of evidences for a detected peptide. ^2^ %Cov (95) indicates the percentage of matching amino acids from identified peptides having confidence greater than or equal to 95, divided by the total number of amino acids in the sequence. ^3^ Confidence assignment for mass spectrometry-based peptide identifications. ^4^ Chemical modifications of identified peptides. Cysteine Carbamidomethylation. ^5^ Missed cleavages sites. ^6^ The difference in mass between the observed molecular weight (MW) and the theoretical MW of the matching peptide sequence. ^7^ The monoisotopic mass for the ion fragmented in this cycle and experiment. ^8^ The monoisotopic mass-to-charge ration (*m/z*) for the ion fragmented in this cycle and experiment. ^9^ The MW of the peptide calculated from the sequence and modifications. ^10^ Theoretical MW/theoretical z. ^11^ For those spectra for which the charge state could not be determined from the data, this is the charge state for the highest scoring peptide. ^12^ The score for the peptide based on matching ions of various charge states. ^13^ Spectrum denotes a particular MS/MS spectrum in this processing run. ^14^ The retention time for this spectrum. ^15^ The elution profile of every peptide identified, fitting the peak shape to a Gaussian function. ^16^ Time required to fragment all ions filling each of the precursor *m/z* in a quadrupole-Orbitrap mass spectrometer. ^17^ Ion intensity measurements of peptide precursors in the MS1 scan. ^18^ Precursor signal intensity. ^19^ Number of spectra per peptides acquired by fragmentation analysis (MS2).

## References

[1] Georgis R., Koppenhöfer A.M., Lacey L.A., Bélair G., Duncan L.W., Grewal P.S., Samish M., Tan L., Torr P., van Tol R.W.H.M. (2006). Successes and failures in the use of parasitic nematodes for pest control. Biol. Control.

[2] Ehlers R.-U. (2001). Mass production of entomopathogenic nematodes for plant protection. Appl. Microbiol. Biotechnol..

[3] Lacey L.A., Georgis R. (2012). Entomopathogenic nematodes for control of insect pests above and below ground with comments on commercial production. J. Nematol..

[4] Dillman A.R., Sternberg P.W. (2012). Entomopathogenic nematodes. Curr. Biol..

[5] Ibrahim E., Dobeš P., Kunc M., Hyršl P., Kodrík D. (2018). Adipokinetic hormone and adenosine interfere with nematobacterial infection and locomotion in Drosophila melanogaster. J. Insect Physiol..

[6] Arefin B., Kucerova L., Dobes P., Markus R., Strnad H., Wang Z., Hyrsl P., Zurovec M., Theopold U. (2014). Genome-wide transcriptional analysis of Drosophila larvae infected by entomopathogenic nematodes shows involvement of complement, recognition and extracellular matrix proteins. J. Innate Immun..

[7] Kunc M., Arefin B., Hyrsl P., Theopold U. (2017). Monitoring the effect of pathogenic nematodes on locomotion of Drosophila larvae. Fly.

[8] Lewis E.E., Gaugler R., Harrison R. (1992). Entomopathogenic nematode host finding: Response to host contact cues by cruise and ambush foragers. Parasitology.

[9] Lewis E.E., Selvan S., Campbell J.F., Gaugler R. (1995). Changes in foraging behaviour during the infective stage of entomopathogenic nematodes. Parasitology.

[10] Lewis E.E., Campbell J., Griffin C., Kaya H., Peters A. (2006). Behavioral ecology of entomopathogenic nematodes. Biol. Control.

[11] Griffin C.T. (2012). Perspectives on the behavior of entomopathogenic nematodes from dispersal to reproduction: Traits contributing to nematode fitness and biocontrol efficacy. J. Nematol..

[12] Campbell J.F., Lewis E.E., Stock S.P., Nadler S., Kaya H.K. (2003). Evolution of host search strategies in entomopathogenic nematodes. J. Nematol..

[13] Shapiro-Ilan D.I., Han R., Dolinksi C. (2012). Entomopathogenic nematode production and application technology. J. Nematol..

[14] Koppenhöfer A.M., Grewal P.S., Fuzy E.M. (2007). Differences in penetration routes and establishment rates of four entomopathogenic nematode species into four white grub species. J. Invertebr. Pathol..

[15] Liao C., Gao A., Li B., Wang M., Shan L. (2017). Two symbiotic bacteria of the entomopathogenic nematode *Heterorhabditis* spp. against Galleria mellonella. Toxicon.

[16] Bode H.B. (2009). Entomopathogenic bacteria as a source of secondary metabolites. Curr. Opin. Chem. Biol..

[17] Rodou A., Ankrah D.O., Stathopoulos C. (2010). Toxins and secretion systems of photorhabdus luminescens. Toxins.

[18] Han R., Ehlers R. (2000). Pathogenicity, development, and reproduction of heterorhabditis bacteriophora and *Steinernema carpocapsae* under axenic in vivo conditions. J. Invertebr. Pathol..

[19] Sicard M., Le Brun N., Pages S., Godelle B., Boemare N., Moulia C. (2003). Effect of native xenorhabdus on the fitness of their steinernema hosts: Contrasting types of interaction. Parasitol. Res..

[20] Ehlers R., Wulff A., Peters A. (1997). Pathogenicity of axenic *Steinernema feltiae*, *Xenorhabdus bovienii*, and the bacto–helminthic complex to larvae of tipula oleracea (Diptera) and galleria mellonella (Lepidoptera). J. Invertebr. Pathol..

[21] Grewal P.S., Peters A. (2005). Formulation and quality. Nematodes as Biocontrol Agents.

[22] Cerenius L., Lee B.L., Söderhäll K. (2008). The proPO-system: Pros and cons for its role in invertebrate immunity. Trends Immunol..

[23] Söderhäll K., Cerenius L. (1998). Role of the prophenoloxidase-activating system in invertebrate immunity. Curr. Opin. Immunol..

[24] Balasubramanian N., Hao Y.-J., Toubarro D., Nascimento G., Simões N. (2009). Purification, biochemical and molecular analysis of a chymotrypsin protease with prophenoloxidase suppression activity from the entomopathogenic nematode *Steinernema carpocapsae*. Int. J. Parasitol..

[25] Hao Y.-J., Montiel R., Nascimento G., Toubarro D., Simoes N. (2009). Identification and expression analysis of the *Steinernema carpocapsae* elastase-like serine protease gene during the parasitic stage. Exp. Parasitol..

[26] Toubarro D., Lucena-Robles M., Nascimento G., Costa G., Montiel R., Coelho A.V., Simões N. (2009). An apoptosis-inducing serine protease secreted by the entomopathogenic nematode *Steinernema carpocapsae*. Int. J. Parasitol..

[27] Balasubramanian N., Toubarro D., Simões N. (2010). Biochemical study and in vitro insect immune suppression by a trypsin-like secreted protease from the nematode *Steinernema carpocapsae*. Parasite Immunol..

[28] Balasubramanian N., Nascimento G., Ferreira R., Martinez M., Simões N. (2012). Pepsin-like aspartic protease (Sc-ASP155) cloning, molecular characterization and gene expression analysis in developmental stages of nematode *Steinernema carpocapsae*. Gene.

[29] Balasubramanian N., Toubarro D., Nascimento G., Ferreira R., Simões N. (2012). Purification, molecular characterization and gene expression analysis of an aspartic protease (Sc-ASP113) from the nematode *Steinernema carpocapsae* during the parasitic stage. Mol. Biochem. Parasitol..

[30] Toubarro D., Avila M.M., Hao Y., Balasubramanian N., Jing Y., Montiel R., Faria T.Q., Brito R.M., Simões N. (2013). A serpin released by an entomopathogen impairs clot formation in insect defense system. PLoS ONE.

[31] Lu D., Macchietto M., Chang D., Barros M.M., Baldwin J., Mortazavi A., Dillman A.R. (2017). Activated entomopathogenic nematode infective juveniles release lethal venom proteins. PLoS Pathog..

[32] Toubarro D., Avila M.M., Montiel R., Simões N. (2013). A pathogenic nematode targets recognition proteins to avoid insect defenses. PLoS ONE.

[33] Toubarro D., Lucena-Robles M., Nascimento G., Santos R., Montiel R., Veríssimo P., Pires E., Faro C., Coelho A.V., Simões N. (2010). Serine protease-mediated host invasion by the parasitic nematode *Steinernema carpocapsae*. J. Biol. Chem..

[34] Jing Y., Toubarro D., Hao Y., Simões N. (2010). Cloning, characterisation and heterologous expression of an astacin metalloprotease, Sc-AST, from the entomoparasitic nematode *Steinernema carpocapsae*. Mol. Biochem. Parasitol..

[35] Kenney E., Hawdon J.M., O’Halloran D., Eleftherianos I. (2019). Heterorhabditis bacteriophora excreted-secreted products enable infection by photorhabdus luminescens through suppression of the imd pathway. Front. Immunol..

[36] Harnett M.M., Kean D.E., Boitelle A., McGuiness S., Thalhamer T., Steiger C.N., Egan C., Al-Riyami L., Alcocer M.J., Houston K.M. (2008). The phosphorycholine moiety of the filarial nematode immunomodulator ES-62 is responsible for its anti-inflammatory action in arthritis. Ann. Rheum. Dis..

[37] Ditgen D., Anandarajah E.M., Meissner K.A., Brattig N., Wrenger C., Liebau E. (2014). Harnessing the helminth secretome for therapeutic immunomodulators. Biomed. Res. Int..

[38] Shepherd C., Navarro S., Wangchuk P., Wilson D., Daly N.L., Loukas A. (2015). Identifying the immunomodulatory components of helminths. Parasite Immunol..

[39] Jančaříková G., Houser J., Dobeš P., Demo G., Hyršl P., Wimmerová M. (2017). Characterization of novel bangle lectin from *Photorhabdus asymbiotica* with dual sugar-binding specificity and its effect on host immunity. PLoS Pathog..

[40] Wagenaar M.M., Gibson D.M., Clardy J. (2002). Akanthomycin, a new antibiotic pyridone from the entomopathogenic fungus akanthomyces gracilis. Org. Lett..

[41] Lee S.-Y., Nakajima I., Ihara F., Kinoshita H., Nihira T. (2005). Cultivation of entomopathogenic fungi for the search of antibacterial compounds. Mycopathologia.

[42] Haydak M.H. (1936). A food for rearing laboratory animals. J. Econ. Entomol..

[43] Rosa J.S., Bonifassi E., Amaral J., Lacey L.A., Simões N., Laumond C. (2000). Natural occurrence of entomopathogenic nematodes (rhabditida: *Steinernema*, heterorhabditis) in the azores. J. Nematol..

[44] Chang D.Z., Serra L., Lu D., Mortazavi A., Dillman A.R. (2019). A core set of venom proteins is released by entomopathogenic nematodes in the genus *Steinernema*. PLoS Pathog..

[45] Bai X., Adams B.J., Ciche T.A., Clifton S., Gaugler R., Kim K., Spieth J., Sternberg P.W., Wilson R.K., Grewal P.S. (2013). A lover and a fighter: The genome sequence of an entomopathogenic nematode heterorhabditis bacteriophora. PLoS ONE.

[46] Shapiro-llan D.I. (2001). Virulence of entomopathogenic nematodes to pecan weevil (Coleoptera: Curculionidae) adults. J. Entomol. Sci..

[47] De Batista E.S.P., Auad A.M., Andaló V., de Monteiro C.M.O. (2014). Virulence of entomopathogenic nematodes (Rhabditida: Steinernematidae, Heterorhabditidae) to spittlebug Mahanarva spectabilis (Hemiptera: Cercopidae). Arq. Inst. Biol..

[48] Koppenhöfer A.M., Grewal P.S., Fuzy E.M. (2006). Virulence of the entomopathogenic nematodes *Heterorhabditis bacteriophora*, *Heterorhabditis zealandica*, and *Steinernema scarabaei* against five white grub species (Coleoptera: Scarabaeidae) of economic importance in turfgrass in North America. Biol. Control.

[49] Del Valle E.E., Frizzo L.S., Lax P., Bonora J.S., Palma L., Bernardi Desch N.P., Pietrobón M., Doucet M.E. (2017). Biological control of *Diloboderus abderus* (Coleoptera: Scarabaeidae) larvae using *Steinernema rarum* CUL (Nematoda: Steinernematidae) and Heterorhabditis bacteriophora SMC (Nematoda: Heterorhabditidae). Crop. Prot..

[50] Crook M. (2014). The dauer hypothesis and the evolution of parasitism: 20 years on and still going strong. Int. J. Parasitol..

[51] Yoder C.A., Grewal P.S., Taylor R.A.J. (2004). Rapid age-related changes in infection behavior of entomopathogenic nematodes. J. Parasitol..

[52] Lee J.H., Dillman A.R., Hallem E.A. (2016). Temperature-dependent changes in the host-seeking behaviors of parasitic nematodes. BMC Biol..

[53] Griffin C., Fitters P. (2004). Spontaneous and induced activity of *Heterorhabditis megidis* infective juveniles during storage. Nematology.

[54] Gaugler R., Campbell J.F., Lewis E.E. (1997). The effects of aging on the foraging behaviour of *Steinernema carpocapsae* (Rhabdita: Steinernematidae). Nematologica.

[55] Leinwand S.G., Yang C.J., Bazopoulou D., Chronis N., Srinivasan J., Chalasani S.H. (2015). Circuit mechanisms encoding odors and driving aging-associated behavioral declines in *Caenorhabditis elegans*. Elife.

[56] Yadav A.K. (2016). Effects of storage temperature on survival and infectivity of three indigenous entomopathogenic nematodes strains (*Steinernematidae* and *Heterorhabditidae*) from Meghalaya, India. J. Parasit. Dis..

[57] Yadav S., Eleftherianos I. (2018). Prolonged storage increases virulence of *Steinernema entomopathogenic* nematodes toward drosophila larvae. J. Parasitol..

[58] Griffin C.T. (1996). Effects of prior storage conditions on the infectivity of Heterorhabditis sp. (Nematoda: Heterorhabditidae). Fundam. Appl. Nematol..

[59] Fitters P.F.L., Dunne R., Griffin C.T. (2001). Improved control of otiorhynchus sulcatus at 9 °C by cold-stored heterorhabditis megidis UK211. Biocontrol Sci. Technol..

[60] Shapiro-Ilan D.I., Hazir S., Lete L. (2015). Viability and virulence of entomopathogenic nematodes exposed to ultraviolet radiation. J. Nematol..

[61] Perez E.E., Lewis E.E., Shapiro-Ilan D.I. (2003). Impact of the host cadaver on survival and infectivity of entomopathogenic nematodes (Rhabditida: *Steinernematidae* and Heterorhabditidae) under desiccating conditions. J. Invertebr. Pathol..

[62] Hominick W.M., Reid A.P. (1990). Perspectives on Entomopathogenic Nematology.

[63] Dempsey C.M., Griffin C.T. (2002). Phased activity in Heterorhabditis megidis infective juveniles. Parasitology.

[64] Campbell J.F., Koppenhöfer A.M., Kaya H.K., Chinnasri B. (1999). Are there temporarily non-infectious dauer stages in entomopathogenic nematode populations: A test of the phased infectivity hypothesis. Parasitology.

[65] Huang C.-C., Hall D.H., Hedgecock E.M., Kao G., Karanzta V., Vogel B.E., Hutter H., Chisholm A.D., Yurchenco P.D., Wadswarth W.G. (2003). Laminin subunits and their role in C. elegans development. Development.

[66] Hollister K.A., Conner E.S., Zhang X., Spell M., Bernard G.M., Patel P., de Carvalho A.C.G.V., Butcher R.A., Ragains J.R. (2013). Ascaroside activity in Caenorhabditis elegans is highly dependent on chemical structure. Bioorg. Med. Chem..

[67] Zhang X., Wang Y., Perez D.H., Jones Lipinski R.A., Butcher R.A. (2018). Acyl-CoA oxidases fine-tune the production of ascaroside pheromones with specific side chain lengths. ACS Chem. Biol..

[68] Noguez J.H., Conner E.S., Zhou Y., Ciche T.A., Ragains J.R., Butcher R.A. (2012). A novel ascaroside controls the parasitic life cycle of the entomopathogenic nematode heterorhabditis bacteriophora. ACS Chem. Biol..

[69] Butcher R.A., Fujita M., Schroeder F.C., Clardy J. (2007). Small-molecule pheromones that control dauer development in Caenorhabditis elegans. Nat. Chem. Biol..

[70] Srinivasan J., von Reuss S.H., Bose N., Zaslaver A., Mahanti P., Ho M.C., O’Doherty O.G., Edison A.S., Sternberg P.W., Schroeder F.C. (2012). A modular library of small molecule signals regulates social behaviors in caenorhabditis elegans. PLoS Biol..

[71] Macosko E.Z., Pokala N., Feinberg E.H., Chalasani S.H., Butcher R.A., Clardy J., Bargmann C.I. (2009). A hub-and-spoke circuit drives pheromone attraction and social behaviour in C. elegans. Nature.

[72] Butcher R.A. (2017). Decoding chemical communication in nematodes. Nat. Prod. Rep..

[73] Alonso V., Nasrolahi S., Dillman A. (2018). Host-specific activation of entomopathogenic nematode infective juveniles. Insects.

[74] Rosa J.S., Simões N. (2004). Evaluation of twenty-eight strains of Heterorhabditis bacteriophora isolated in Azores for biocontrol of the armyworm, Pseudaletia unipuncta (Lepidoptera: Noctuidae). Biol. Control.

[75] Vadnal J., Ratnappan R., Keaney M., Kenney E., Eleftherianos I., O’Halloran D., Hawdon J.M. (2017). Identification of candidate infection genes from the model entomopathogenic nematode Heterorhabditis bacteriophora. BMC Genom..

[76] Hao Y.-J., Montiel R., Lucena M.A., Costa M., Simoes N. (2012). Genetic diversity and comparative analysis of gene expression between Heterorhabditis bacteriophora Az29 and Az36 isolates: Uncovering candidate genes involved in insect pathogenicity. Exp. Parasitol..

[77] Eleftherianos I., Revenis C. (2011). Role and importance of phenoloxidase in insect hemostasis. J. Innate Immun..

[78] Smith V.J. (2010). Immunology of Invertebrates: Cellular. Encyclopedia of Life Sciences.

[79] Schmidt O., Theopold U., Strand M. (2001). Innate immunity and its evasion and suppression by hymenopteran endoparasitoids. BioEssays.

[80] Ligoxygakis P. (2002). A serpin mutant links Toll activation to melanization in the host defence of Drosophila. EMBO J..

[81] Park J.-W., Kim C.-H., Kim J.-H., Je B.-R., Roh K.-B., Kim S.-J., Lee H.-H., Ryu J.-H., Lim J.-H., Oh B.-H. (2007). Clustering of peptidoglycan recognition protein-SA is required for sensing lysine-type peptidoglycan in insects. Proc. Natl. Acad. Sci. USA.

[82] Großhans J., Schnorrer F., Nüsslein-Volhard C. (1999). Oligomerisation of tube and pelle leads to nuclear localisation of dorsal. Mech. Dev..

[83] Huot L., George S., Girard P.-A., Severac D., Nègre N., Duvic B. (2019). Spodoptera frugiperda transcriptional response to infestation by *Steinernema carpocapsae*. Sci. Rep..

[84] Ji S., Sun M., Zheng X., Li L., Sun L., Chen D., Sun Q. (2014). Cell-surface localization of Pellino antagonizes Toll-mediated innate immune signalling by controlling MyD88 turnover in Drosophila. Nat. Commun..

[85] Haghayeghi A., Sarac A., Czerniecki S., Grosshans J., Schöck F. (2010). Pellino enhances innate immunity in Drosophila. Mech. Dev..

[86] Cluxton C.D., Caffrey B.E., Kinsella G.K., Moynagh P.N., Fares M.A., Fallon P.G. (2015). Functional conservation of an ancestral Pellino protein in helminth species. Sci. Rep..

[87] Davies B.A., Topp J.D., Sfeir A.J., Katzmann D.J., Carney D.S., Tall G.G., Friedberg A.S., Deng L., Chen Z., Horazdovsky B.F. (2003). Vps9p CUE domain ubiquitin binding is required for efficient endocytic protein traffic. J. Biol. Chem..

[88] Zhu L.-L., Luo T.-M., Xu X., Guo Y.-H., Zhao X.-Q., Wang T.-T., Tang B., Jiang Y.-Y., Xu J.-F., Lin X. (2016). E3 ubiquitin ligase Cbl-b negatively regulates C-type lectin receptor–mediated antifungal innate immunity. J. Exp. Med..

[89] Theopold U., Rissler M., Fabbri M., Schmidt O., Natori S. (1999). Insect glycobiology: A lectin multigene family in drosophila melanogaster. Biochem. Biophys. Res. Commun..

[90] Yu X.-Q., Gan H.R., Kanost M. (1999). Immulectin, an inducible C-type lectin from an insect, Manduca sexta, stimulates activation of plasma prophenol oxidase. Insect Biochem. Mol. Biol..

[91] Yu X.-Q., Kanost M.R. (2000). Immulectin-2, a lipopolysaccharide-specific lectin from an insect, manduca sexta, is induced in response to gram-negative bacteria. J. Biol. Chem..

[92] Kud J., Wang W., Gross R., Fan Y., Huang L., Yuan Y., Gray A., Duarte A., Kuhl J.C., Caplan A. (2019). The potato cyst nematode effector RHA1B is a ubiquitin ligase and uses two distinct mechanisms to suppress plant immune signaling. PLoS Pathog..

[93] Chronis D., Chen S., Lu S., Hewezi T., Carpenter S.C.D., Loria R., Baum T.J., Wang X. (2013). A ubiquitin carboxyl extension protein secreted from a plant-parasitic nematode *Globodera rostochiensis* is cleaved in planta to promote plant parasitism. Plant. J..

[94] Eves-van den Akker S., Laetsch D.R., Thorpe P., Lilley C.J., Danchin E.G.J., Da Rocha M., Rancurel C., Holroyd N.E., Cotton J.A., Szitenberg A. (2016). The genome of the yellow potato cyst nematode, *Globodera rostochiensis*, reveals insights into the basis of parasitism and virulence. Genome Biol..

[95] Chen C., Cui L., Chen Y., Zhang H., Liu P., Wu P., Qiu D., Zou J., Yang D., Yang L. (2017). Transcriptional responses of wheat and the cereal cyst nematode *Heterodera avenae* during their early contact stage. Sci. Rep..

